# The Feasibility and Early Results of Multivessel Minimally Invasive Coronary Artery Bypass Grafting for All Comers

**DOI:** 10.3390/jcm12175663

**Published:** 2023-08-31

**Authors:** Ergun Demirsoy, Ilhan Mavioglu, Emre Dogan, Harun Gulmez, Ismet Dindar, Mustafa Kemal Erol

**Affiliations:** 1 Division of Cardiovascular Surgery, Sisli Kolan International Hospital, Kaptanpaşa Mahellesi Darulaceze Caddesi No 14, Sisli, 34384 Istanbul, Turkey; 2 Division of Cardiovascular Surgery, Private Practice, Sisli, 34394 Istanbul, Turkey; maviogluilhan@gmail.com; 3 Division of Cardiology, Sisli Kolan International Hospital, 34384 Istanbul, Turkey

**Keywords:** coronary artery bypass grafting, off-pump coronary artery bypass, minimally invasive surgical procedures, stroke, obesity, contraindications

## Abstract

Objectives: Cardiovascular surgery advancements have emerged with various minimally invasive approaches for treating multivessel coronary disease to improve outcomes and minimize the burden associated with conventional cardiac surgery. We present our clinical experience and minimally invasive coronary bypass techniques through minithoracotomy, which we apply without selection to patients who have decided to have elective surgery for multivessel isolated coronary artery disease. Methods: It consists of 230 consecutive patients operated by the same team with this method between July 2020 and September 2022. The patients were assigned to one of the two methods preoperatively to their accompanying comorbidities and operated on either with blood cardioplegia via 5 to 7 cm left anterior minithoracotomy, with on-pump clamped technique or without pump via left anterolateral minithoracotomy. Results: Mortality was observed in two of our patients (0.9%), but myocardial infarction was not observed in our patients in the early postoperative period. None of our patients required conversion to sternotomy (0%). Five patients’ needed reoperation from the same incision due to postoperative bleeding (2.2%), and atrial fibrillation developed in 17 patients in the postoperative period (7.4%). The mean number of bypasses was found to be 3.0 ± 0.9. Conclusions: Minimally invasive coronary artery bypass surgery via minithoracotomy can be routinely reproduced safely. More long-term results and more multicenter studies are needed for more widespread acceptance of the technique.

## 1. Introduction

Successful coronary artery bypass grafting (CABG) surgery results in the classical on-pump clamped bypass technique, which has been the gold standard for many years. Nevertheless, there are some drawbacks to this CABG surgery. The most important ones are nonunion, healing problems, and deep wound infection of the sternum. Although bilateral ITA is avoided, especially in obese female patients, with the foresight that using BITA grafts will impair sternal wound healing, the sternal wound healing problem is seen in nearly 10% of CABG patients [1]. Deep sternal wound infection (DSWI) is the most devastating complication of the sternotomy performed for CABG. DSWI, if the sternum and mediastinal space are contained in the infection, is less common than a superficial one but is a major complication after sternotomy, with a reported incidence of between 0.25% and 5.0% [2]. The Swedeheart study published in 2021 showed that the presence of DSWI significantly increased both short-term and long-term mortality rates [3]. Another important issue is the time needed to return to everyday life after CABG. Studies conducted on patients undergoing CABG have shown that it may take 3–6 months for physical activity to return to normal, and for most patients, it may take up to 1 year for them to return to everyday life [4].

Despite successful results of classical CABG, these things have led to the search for various minimal approaches. Bonatti et al. reviewed nearly all minimally invasive techniques in their published study [5]. First, a minimally invasive left internal thoracic artery (LITA) to the left anterior descending (LAD) bypass on a beating heart was published in 1996 [6]. Avoiding sternal bone healing problems and the short recovery period made minimally invasive approaches attractive. Since then, numerous reports have reported techniques and minimally invasive coronary artery bypass (MICAB) results. Despite many centers having published feasible and successful results of MICAB, even in obese patients, it has not gained widespread prevalence due to its biting and somewhat long learning curve and various relative or definite contraindications [7,8,9,10]. It has been reported in the literature that despite a definite learning curve for MICAB, there is no problem even with this learning curve, a decrease in operation times and the rate of conversion to sternotomy, and an increase in the number of bypasses over time [8,9]. Technical difficulties (femoral cannulation, distance to heart, etc.) such as a relative or absolute contraindication in patients with severe peripheral arterial disease, chronic obstructive pulmonary disease, morbid obesity, or bone abnormalities, and the thought that surgical technique is complex have prevented it from becoming widespread. Even the fact that it has been shown in various recent articles that the long-term results are not different from the classical sternotomy methods have not led to its widespread use [11,12,13].

We present the MICAB method to all consecutive patients who apply for surgical treatment of isolated coronary artery disease without considering any contraindication. MICAB is performed routinely with the modified on-pump clamped multivessel MIDCAB technique for all consecutive patients requiring isolated coronary artery bypass grafting or the minimally invasive off-pump anaortic coronary artery bypass (MACAB) technique [14,15] for those with porcelain aorta, peripheral artery disease, and chest deformities, etc.

## 2. Methods

IRB (Institutional Review Board) approval was granted for this study protocol from the International Kolan Hospital, number 0156, on 25 April 2022. Written informed consent was obtained from each patient to include their information in this manuscript. The files of 230 consecutive patients who underwent isolated coronary artery bypass surgery since May 2020 were retrospectively reviewed for baseline characteristics, surgical data, and postoperative outcomes. Redo cases, emergent cases (a total of 5 emergent cases belong to the same interval), and patients who underwent additional procedures were excluded. Five patients classified as emergent with hemodynamically unstable acute myocardial infarction during the study were excluded because they were operated on using sternotomy. Continuous variables are expressed as mean ± standard deviation, and categorical variables are expressed as numbers and frequencies (percentages) for statistical analysis.

### 2.1. Preoperative Preparation and Operative Technique

In addition to routine preparations, previous chest-related surgeries and diseases should be questioned in detail. Preoperative tomographic angiography or detailed Doppler ultrasonography should demonstrate that peripheral vessels are safe and unproblematic for cannulation. Bone deformities, possible thoracotomy range, the distance of ITA, the depth of the heart, and the length of surgical instruments can be predicted by transforming tomographic images into three dimensions (3D).

Individuals who have a heavily calcified ascending aorta and moderate to severe chronic obstructive pulmonary disease, severe peripheral artery disease, borderline renal disorder, and severe deformities were preoperatively assigned to the off-pump anaortic technique according to the results of the preoperative evaluation. The MACAB (off-pump, anaortic) technique has been described in detail in previously published articles [14,15,16]. The method described here is the minimally invasive coronary artery bypass with an on-pump and clamped technique.

#### Perioperative Details (Video)

**Figure 1:**
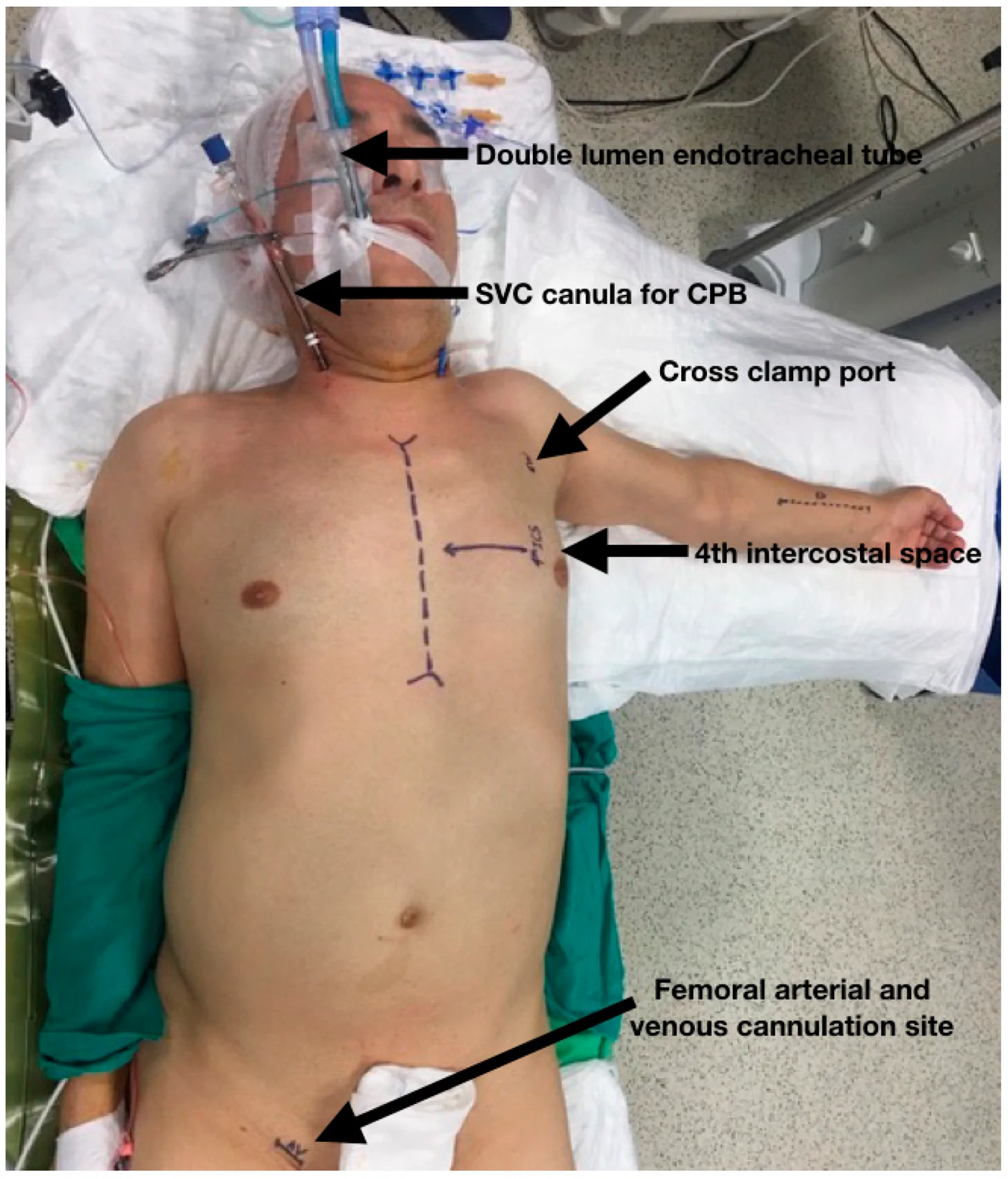
Double lumen endotracheal tube and SVC cannula in place can be seen, and before the patient was stained, markings for the intercostal spaces, the location of the incisions, and the port for cross-clamp to be used were made.

Double lumen endotracheal intubation was applied to ensure left lung isolation. Transesophageal echocardiography (TEE) was used to rule out any cardiac abnormalities before and to control hemodynamic status during operation. The semi-supine position was provided, and the left chest was elevated to the desired extent via a pressure cuff. Before the incision, the skin is marked with a surgical pen for proper incision enlargement in an emergency (Figure 1). All patients must have right jugular venous cannulation to facilitate venous drainage during CPB. The left or right femoral region was prepared according to preop CT and included in the sterile field.

The left radial artery (LRA) is harvested by using endoscopic vessel harvesting (EVH) system (Vasoview Hemopro EVH System, Getinge, Wayne, NJ, USA) and then skeletonized proximally to ease of use during end-to-side anastomoses when creating a y-graft with the LITA. The endoscopic vessel harvesting (EVH) technique was preferred for harvesting all saphenous veins and radial arterial grafts. Additionally, all the grafts were marked with a surgical pen to prevent twisting and torsion.

A small oblique incision 2.5 cm long is made in the groin. The femoral artery and vein are prepared for cannulation. After heparin administration, vascular cannulation is initiated for CPB. The right axillary artery was used for arterial inflow due to one patient’s poor femoral vessel quality.

Left anterior thoracotomy incision 5 to 7 cm long through the fourth intercostal space is performed; in women, a submammary crease incision is preferred. In most cases, thoracotomy is performed through the 4th intercostal space. Various retractor systems have been developed to harvest the LITA or BITA. After placing the retractor system (MIDAccess IMA Retractor, Delacroix-Chevalier, Paris, France), the port for the endocamera is inserted in the midaxillary line in the 3rd intercostal space without overlapping.

There are advantages to using an endocamera with a 30-degree angle during ITA preparation. Unlike the direct view, the surgeon can view the ITA from different directions and angles throughout its course. Since the images are 2D, it is a disadvantage that requires adaptation and an individual learning curve for hand-eye coordination [17]. Advanced 3D images provided by the 3D endocameras and screens will likely enhance the performance [18,19].

Electrocautery is commonly used to harvest the LITA. ITA harvesting in a skeletonized fashion using a harmonic scalpel (Ethicon Endo-Surgery, CVD, Cincinnati, OH, USA) was published as a safe and quick technique [20]. However, the LigaSure (Covidien Products, Medtronic, Minneapolis, MN, USA) and the Maryland Jaw tip are preferred for sealing ITA branches during thoracotomy harvesting [21].

**Figure 2:**
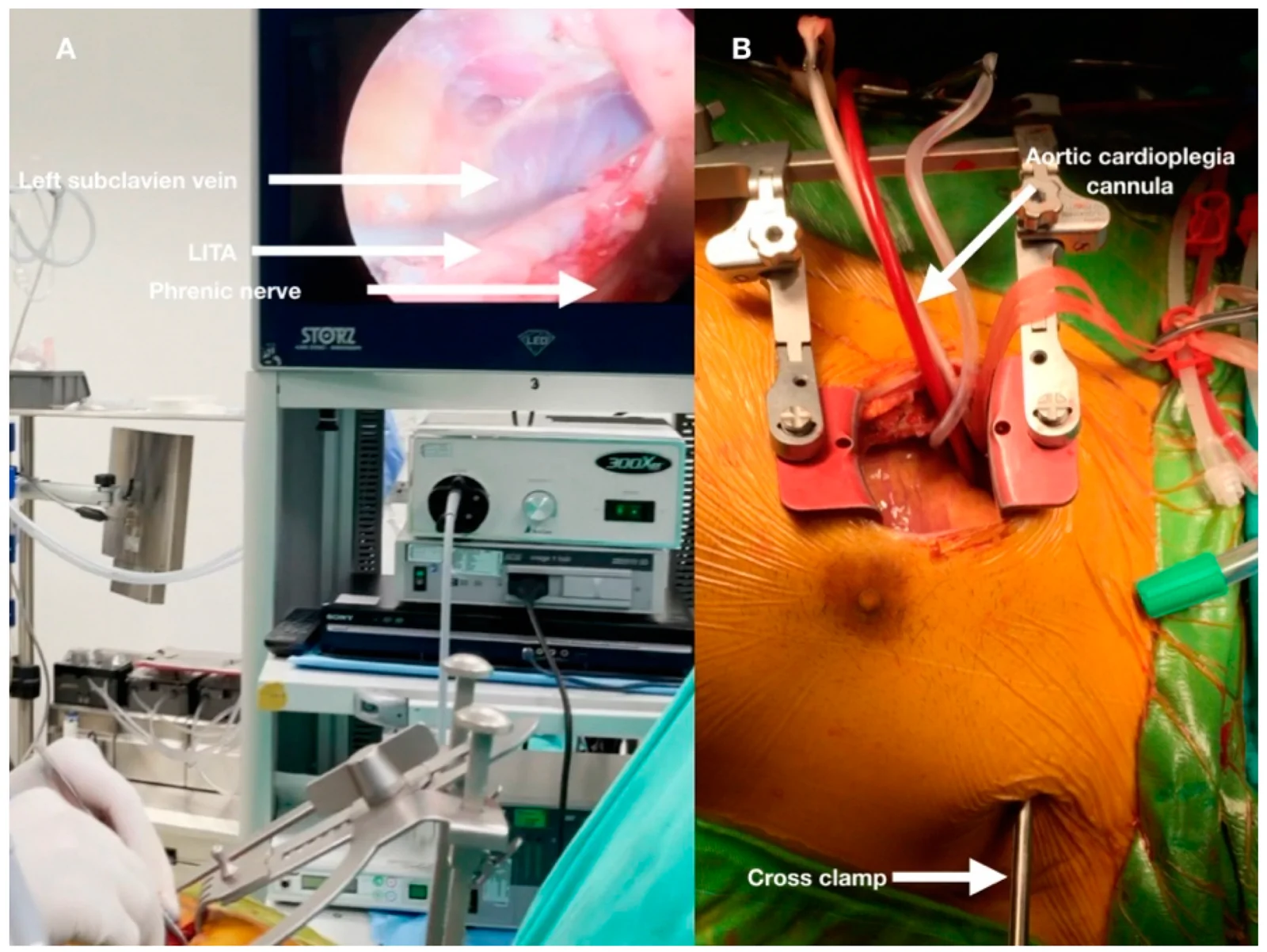
(**A**) The images obtained from the camera during the proximal harvesting of the LITA are incredibly beneficial in preventing phrenic nerve injury. (**B**) The heart stops in diastole during isothermal blood cardioplegia delivery from the ascending aorta after Chitwood cross-clamping.

The anterior thoracic fascia can be divided with electrocautery or scissors. The skeletonized technique was preferred for LITA harvesting using a LigaSure Maryland Jaw for sealing and dividing the side branches. The LITA is harvested with standard instruments, skeletonized to its full length beyond the subclavian vein. The LITA length is nearly sufficient for all cases to reach the target vessel. Care should be taken not to injure the phrenic nerve, especially when approaching the subclavian vein (Figure 2A).

Cardiopulmonary bypass was started at the end of harvesting. Vacuum-assisted venous return-improving heart decompression was routinely used during CPB; most patients were kept normothermic during CPB. The pericardium was opened until the ascending aorta, parallel to the course of the LAD, 2–3 cm above the phrenic nerve. The base of the pericardial incision at the apex is extended anteriorly and laterally to the phrenic nerve in an inverted “T” shape, like a sternotomy case.

The ascending aorta was encircled with 6 mm of Dacron tape for controlling position. The Dacron tape is slightly pulled to the left side to ease cardioplegia cannula insertion, and the cannula is secured with purse-string sutures. A Chitwood-type DeBakey cross-clamp is inserted through the opening at the anterior axillary line of the second intercostal space to clamp the ascending aorta, and isothermic blood cardioplegia is then started (Figure 2B). The isothermic blood cardioplegia is repeated after each distal anastomosis (through a saphenous or radial artery graft) or every 20 min.

Dacron tapes encircle the left pulmonary veins, and the inferior vena cava are used to hang the heart to expose target lesions for anastomosis. After pulling the ascending aorta downward with Dacron tape to close the aorta to the incision site for proximal anastomosis, the aortic side clamp is inserted. The surgical technique described in the reference article was used in patients who underwent the off-pump procedure [14,15]. The pericardium is partially closed with separate sutures using mediastinal fat to prevent dislodgement of the heart to the left chest cavity. The straight chest tube is placed in the thoracic cavity.

## 3. Results

**Figure 3:**
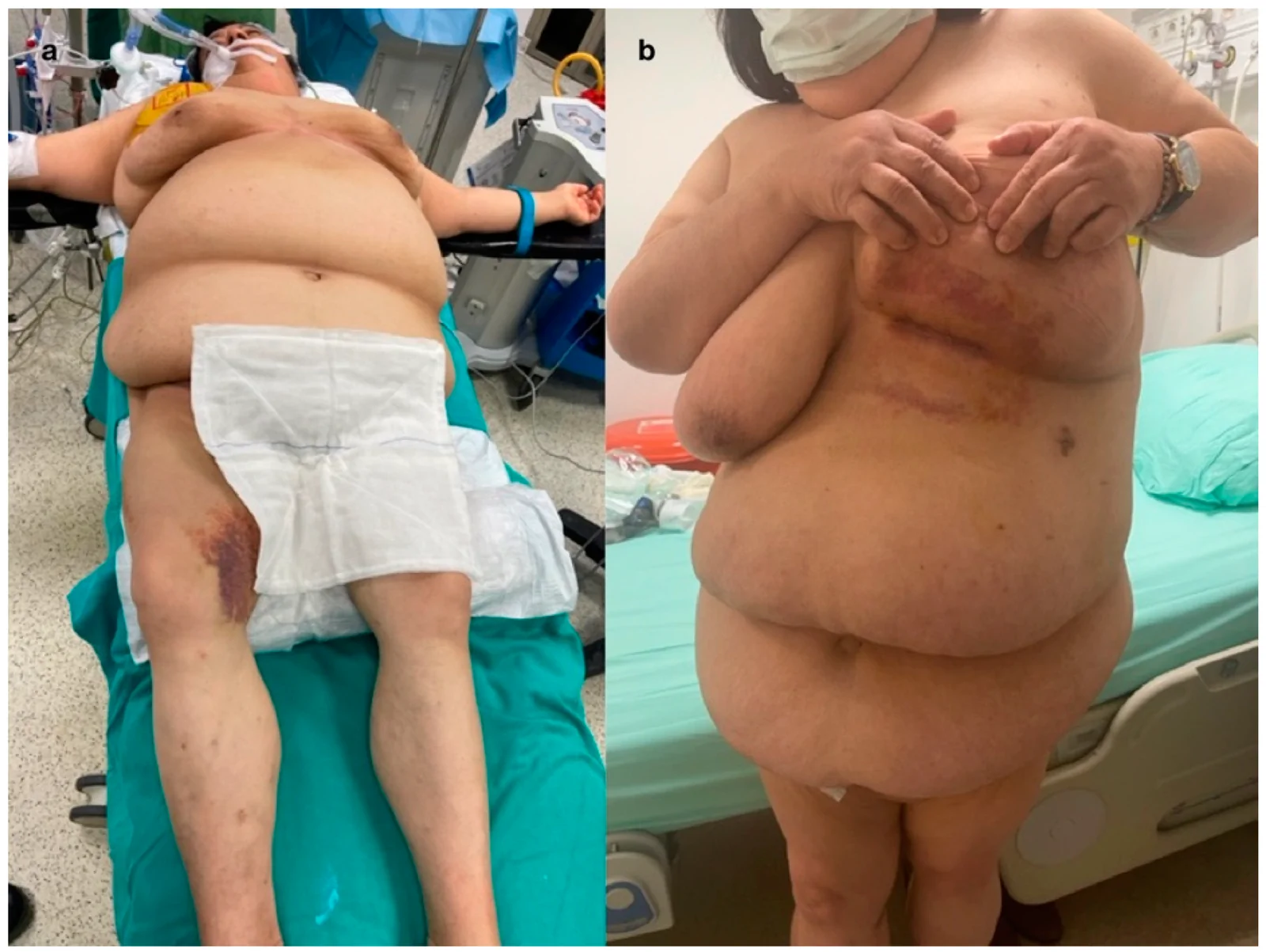
Female patient with morbid obesity. (**a**) On the operating table before surgery; (**b**) seen standing on the 4th postoperative day.

**Figure 4:**
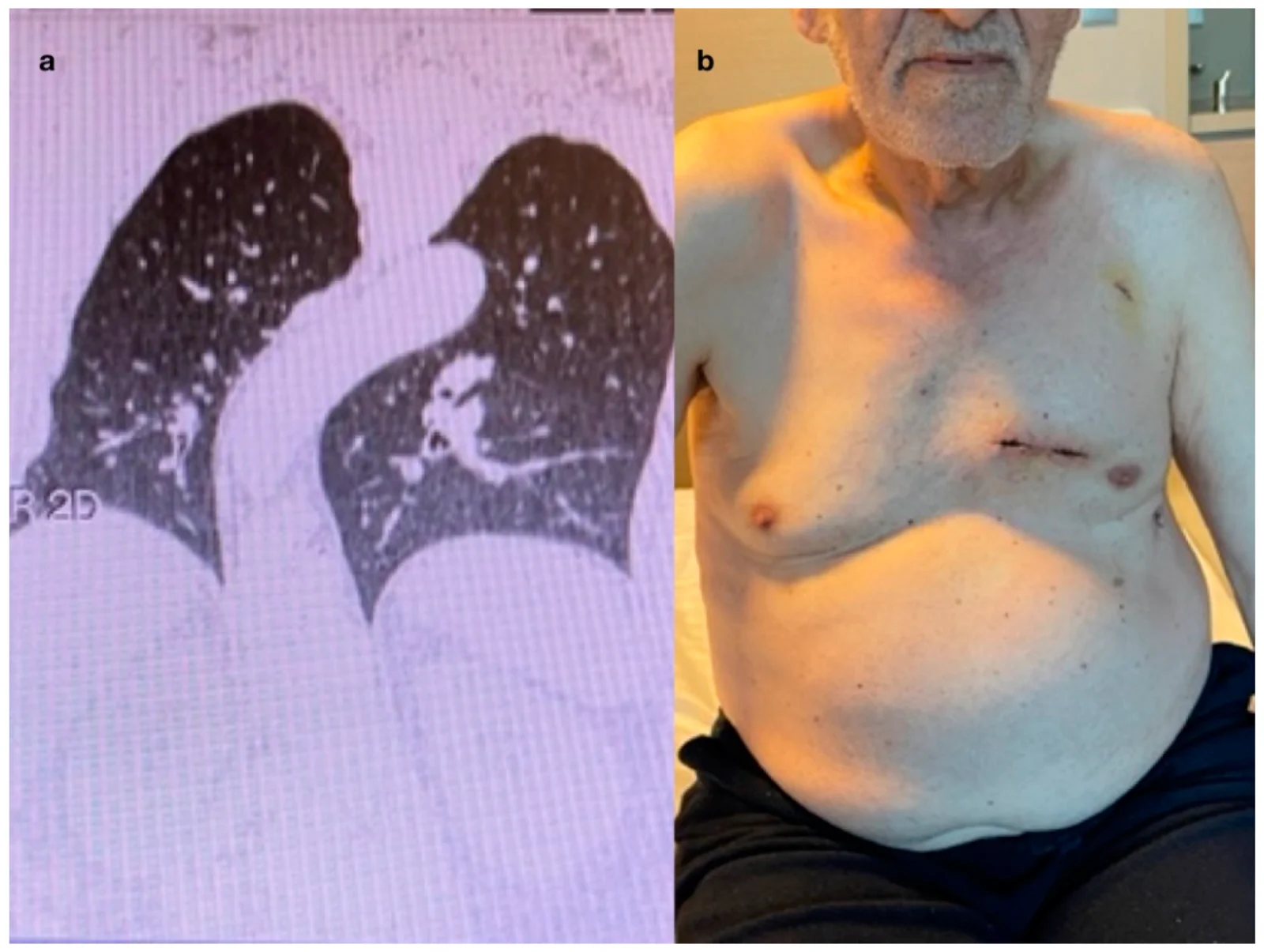
(**a**) A coronal section from the preoperative CT of the patient with severe kyphoscoliosis, and (**b**) the wound in the first week postoperatively can be seen.

Between July 2020 and September 2022, 230 MICAB operations were performed in our institution by the same surgeon (ED). The preoperative findings and associated comorbidities of the patients are provided in Table 1. Nearly 30% of the patients had a BMI greater than 30, and some had morbid obesity (Figure 3a,b). Two of these obese patients had had bariatric surgery before. Three patients (1.3%) had previously been operated on for lung cancer; right pneumectomy had been performed in one, and right and left lung lobectomy had been conducted in the others. Interestingly, one of our patients had very severe kyphoscoliosis (Figure 4a,b), and another had had surgery for scoliosis. During the thoracotomy, apart from adhesions, no problem was encountered that would hinder the operation.

Three or more distals were performed in 70.0% of the patients, and the mean number of bypasses was 3.0 ± 0.9 (Table 2). In 20% of patients, the bypasses were performed off-pump with arterial grafts using the MACAB technique. In all patients using the off-pump technique, a composite graft was created by anastomosing the radial artery to in situ LITA in a “y” or “t” fashion to match the angle of the next sequential anastomosis. Using LITA as a composite graft with the radial artery allowed for bypassing of all targets. Except for using radial artery and anaortic techniques in all off-pumped cases, no difference was found in bypassing all targets compared to those we had on-pumped. Complete revascularization was achieved in all patients included in this study.

**Figure 5:**
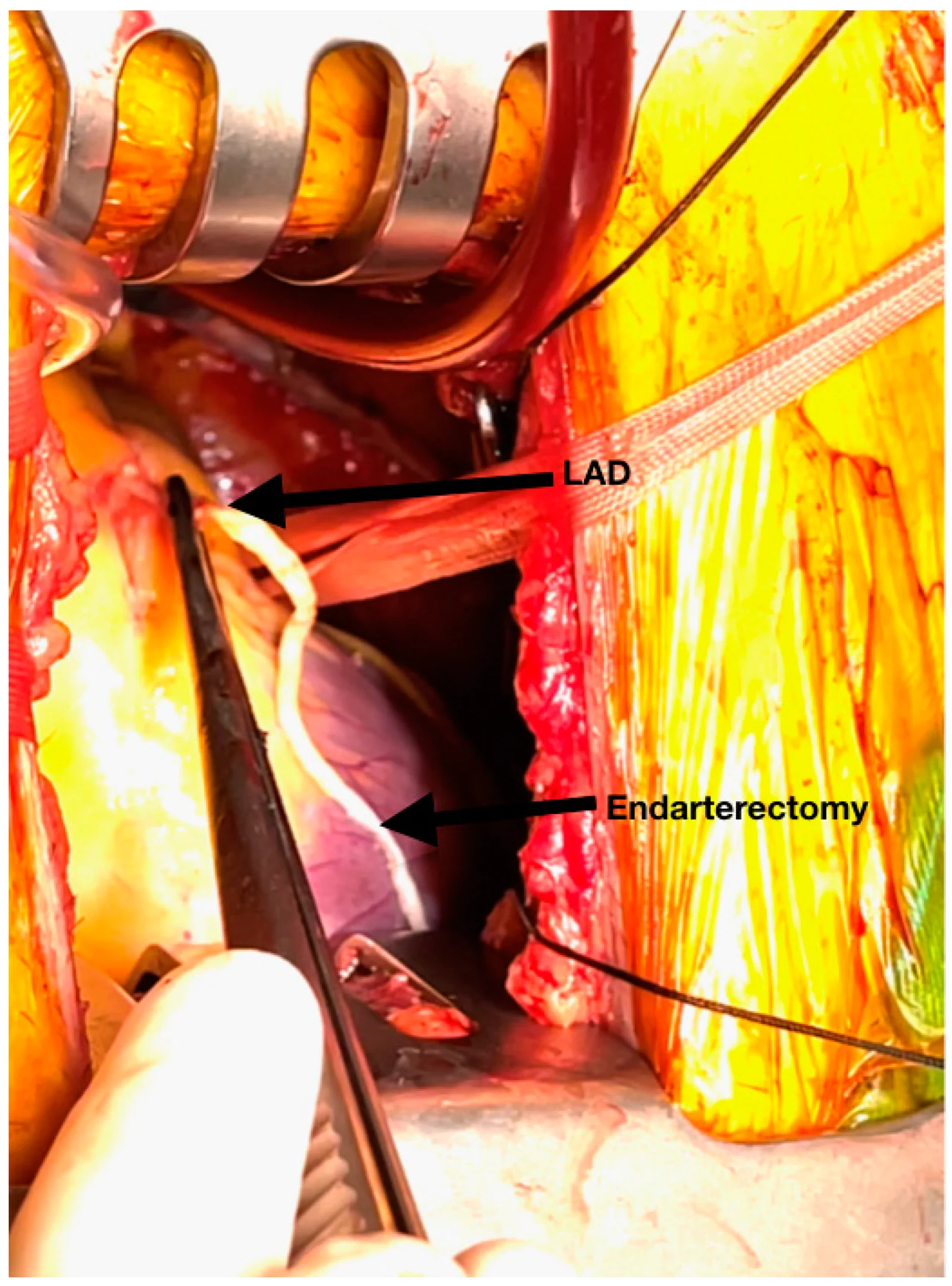
Nearly 8 cm long endarterectomy material extracted from the arteriotomy on the LAD can be seen.

The LITA was used in almost all patients (98%), and as a second artery graft, 38% used the LRA and, recently, the RITA. There was no obstacle in successfully applying endarterectomy through thoracotomy to three patients who required it (Figure 5).

The total operation time was 4.3 ± 1.0 h, CPB time was 151 ± 45.4 min, and aortic cross-clamp time was 78 ± 22.4 min. Postoperative outcomes are summarized in Table 3. Mortality (0.9%) was observed in one patient who was operated on in the prodromal period and developed postoperative pneumonia due to COVID-19 and subsequently intractable respiratory failure, and in another patient, possibly due to acute aortic dissection that developed from the cross-clamp or side-clamp area.

Five patients (2.2%) were returned to the operating room for bleeding control during postoperative follow-ups. Postoperative bleeding revision was performed through the same incision in all patients. In two of them, clips were applied to the bleeding foci formed from the side branches of the grafts. No bleeding focus was observed in the other three patients, and it was decided that the bleeding was due to the use of direct oral anticoagulants (DOACs) in the preoperative period.

Stroke was seen in three patients (1.3%) and resolved without major neurological deficit before discharge from the hospital. Although new-onset AF was seen in 17 patients (7.5%), it was thought that it was seen so rarely due to the use of beta-blockers starting from the preoperative period in almost all patients. In addition, applying more effective postoperative respiratory physiotherapy in patients who underwent thoracotomy compared to sternotomy may have reduced the development of AF by reducing hypoxia rates.

In the early stages of the learning curve, phrenic nerve palsy was observed in two patients (0.9%). Care is required to prepare the proximal section of the LITA near the left subclavian vein. The use of endoscopic cameras is of great importance in preventing this complication (Figure 2A).

One patient (0.4%) experienced chylothorax, which resolved with dieting and pleural drainage with the valve system. Superficial thoracic infection was seen in four patients (1.8%). One was treated with negative pressure wound therapy (V.A.C. Therapy, 3 M), and the others were treated with local dressing. The stay in the intensive care unit was 1.5 ± 1.3 days. The total hospital stay was 5.5 ± 2.2 days.

Coronary angiography was performed on 12 patients who were symptomatic in the early postoperative period. Since the saphenous vein graft was not visualized in four (1.75% early occlusion), a stent was applied to the lesions in the native coronary vessel.

Postoperative follow-up of the patients continues. A study including surveillance with CT angiography was planned for postoperative follow-up, and patient registration was started.

## 4. Discussion

This retrospective observational study shows that surgery can be performed with minimally invasive techniques for all patients, including those with extreme conditions, regardless of patient selection for isolated coronary artery bypass. MICAB can be performed using an on-pump in most patients or off-pump and no touch to aorta techniques in patients with a porcelain aorta, severe peripheral artery disease, or high risk for CPB.

The mortality of 0.9% found in our study is far beyond the reported mortality rates in contemporary studies. Comparing the hospital mortality values of 101,188 patients who underwent CABG in the STS database published in 2011 with a publicly available national death registry database (SSDMF) maintained by the United States Social Security Administration, 30-day mortality rates ranged from 1.1% to 5.3% [22]. Similarly, the 30-day mortality rate for off-pump and on-pump conventional coronary bypass surgery was reported as 1.5% by the ISMICS consensus conference held in 2015 after they reviewed the large prospective randomized studies’ evaluations [23].

The reliability of the new surgical technique must depend mostly on its safety when performed. This is why critical maneuvers must be performed on-pump with a decompressed heart and decreased mean pressure at the aorta, such as encircling the ascending aorta with tape. Similarly, the tapes that encircle the LPVs and IVC are passed on after cross-clamping and cardioplegia, where the heart is on diastolic arrest, empty, and flaccid. This increases the safety and eases manipulation with minimum trauma to the adjacent tissues.

Puskas et al. reported rates of 2.3% and 2.6% for postoperative bleeding revision in off-pump and on-pump coronary bypass surgery, respectively, when 19,101 patients were evaluated in 102 prospective randomized studies reviewed at the ISMICS consensus conference [23]. In MICAB surgery, slightly lower (2.19%) rates for return to the operation room due to bleeding were seen in our series. More experienced surgeons perform MICAB surgery so that hemostasis may be more effective. In addition, using harmonic devices to harvest ITA in these series may reduce postoperative bleeding [24].

Wound infection after MICAB is limited to the minithoracotomy area and follows a different course, with less significant consequences, than DSWI and mediastinitis. The ISMICS consensus conference reported the infection rates for off-pump and on-pump coronary bypass surgery in the range of 2.4% to 4.4%, respectively [23]. The thoracic infection rate of 1.75% after MICAB surgery was somewhat lower than the reported ones. MICAB may be of advantage for insulin-dependent diabetics, obese female patients, and those with COPD, where the risk of mediastinitis is significantly higher, especially with the use of bilateral ITA. Seroma and local infection in the femoral cannulation area, which we observed in two of our patients (0.88%), is one of this method’s feared and annoying complications. To avoid this complication, it is crucial to make the incision for cannulation in the femoral region obliquely from a higher level than the classical place and to control meticulous bleeding.

A widely accepted definition of minimally invasive coronary surgery will facilitate better expression and future result comparisons [25]. In this definition, the technique performed without touching the aorta, which significantly reduces the incidence of stroke, among other elements, should be remembered [26]. Today, the successful performance of completely endoscopic coronary artery bypass surgeries such as off-pump with the help of robots will become an increasing trend with the addition of new robotic platforms [27]. MICAB may be an important option for the patients of our colleagues who cannot access these opportunities until robotic platforms and equipment for bypass surgery are more accessible.

Transit time flow measurement (TTFM) and epicardial ultrasound (ECUS) are used for intraoperative bypass graft evaluation on the adequacy of graft function [28]. Combining these two modalities that provide quality assurance (QA) is accepted as the “state-of-the-art” intraoperative graft evaluation [29]. TTFM and ECUS used during CABG surgery have become widely accepted standards [29]. Unfortunately, these evaluations, the major shortcomings of our series, could only be made in very few of our patients due to reimbursement problems.

The demonstration that using second or more arterial grafts in addition to the LITA increases long-term patency rates and life expectancy compared to using the LITA alone has changed our strategy to use more arterial grafts [30,31]. Although more saphenous vein grafts were used in the first part of the series, we have increasingly started using the radial artery (38%) prepared with EVH and, recently, the RITA, especially in young patients. Since the radial artery is recommended as the second best graft in addition to the LITA in many articles [32,33,34], we prefer the radial artery as the second graft both in on-pump cases and in anaortic off-pump cases.

Although it has been shown that those who use simulation systems benefit, quality control is not fully widespread [35]; we think that it will become more common in the future in learning and practicing minimally invasive cardiac surgery. We conclude that training with simulation systems and perhaps the development of coronary surgery as a separate subspecialty may assist in wider adoption of the technique.

It must be remembered that MICAB operation continues to be more challenging than the classical CABG, and there are still controversies about which strategy is more beneficial for the patient. Despite the disadvantages of longer total operative time and CPB duration than in classical sternotomy patients, the much smaller surgical area, better preservation of bone integrity and respiratory physiology, and reduced postoperative bleeding led to more critical advantages than sternotomy. With the increase in the number of cases for the whole team, we hope the times can be shortened even more, as shown in many previous studies. The entire series in this study is an observational study of patients operated on by a single experienced surgeon. Therefore, patient selection bias is possible, and outcomes may likely have been affected by individual learning curves and cannot be universalized. Since it is a new technique, the need to return to larger incisions or sternotomy arising from the technique must be emphasized. The achievability and perioperative safety of the minimally invasive technique applied with left minithoracotomy to all patients without selection were represented in this study. More studies and randomized trials may be necessary to discuss efficacy and wide availability.

## 5. Conclusions

Robotics and minimally invasive techniques are indispensable parts of contemporary surgical coronary revascularization to combat PCI-based interventions in various treatment modalities for coronary artery disease.

There is an exact learning curve, and it necessitates prior training despite an MICAB not being a technologically complex procedure. The encouraging results of the MICAB emphasize the need for forward growth of this method. Despite prolonging operation time, this method deserves further investigation, as it imposes less impact and a lower load on the thoracic tissue than sternotomy approaches. More studies are needed to show long-term outcomes, including computerized tomographic analysis, which will include the results of our patients.

Minimally invasive coronary artery bypass could determine the future of coronary surgery by reducing surgical trauma and may be a good alternative for multivessel stenting. MICAB can be performed even in patients typically excluded from a minimally invasive approach (e.g., morbid obesity, severe scoliosis, previous lung resection, a severe peripheral arterial disease requiring alternative cannulation sites) with satisfactory early postoperative outcomes.

## Figures and Tables

**Table 1:** Preoperative findings and associated comorbidities.

	*n* (Percentage %)
Mean age (y) (range)	60.17 ± 10.3
Female	24 (10.4%)
BMI > 30	28.7 ± 4.4
Chronic obstructive lung disease	136 (59.1%)
Smoking	133 (57.8%)
Hyperlipidemia	136 (59.1%)
Hypertension	147 (63.9%)
Diabetes mellitus	147 (63.9%)
Cancer under remission	9 (3.9%)
Patients on hemodialysis	4 (1.7%)
Peripheral vascular disease	39 (17.0%)
Porcelain aorta	16 (7.0%)
Cerebrovascular disease	10 (4.3%)
Previous myocardial infarction	86 (37.4%)
EF (mean ± SD)	53.4 ± 9.6
Low EF < 30	14 (6.1%)
EF < 50	88 (38.3%)
Left main coronary artery disease	9 (3.9%)

y: year; *n*: number; BMI: body mass index; EF: ejection fraction; SD: standard deviation.

**Table 2:** Operative characteristics.

	*n* (%)
Bypass number (mean ± SD)	3.0 ± 0.9
Single bypass	18 (7.8%)
Double bypass	51 (22.2%)
Triple bypass	90 (39.1%)
Quadruple bypass	65 (28.3%)
Quintet bypass	6 (2.6%)
LITA	225 (97.8%)
RA	88 (38.2%)
RITA	2 (0.9%)
RCA bypass	138 (60.0%)
Sequential	83 (36.1%)
Off pump	47 (20.4%)
On pump	183 (79.6%)
CPT (mean ± SD)	151 ± 45.4
CCT (mean ± SD)	78 ± 22.4
Total operation time (mean ± SD)	4.3 ± 1.0
Conversion to sternotomy	0 (0%)
IABP	2 (0.9%)

*n*: number; SD: standard deviation; LITA: left internal thoracic artery; RA: radial artery; RITA: right internal thoracic artery; RCA: right coronary artery; CPT: cardiopulmonary bypass time; CCT: cross-clamp time; IABP, intraaortic balloon pump.

**Table 3:** Postoperative complications and mortality.

	*n* (%)
Early mortality (postop < 30 days)	2 (0.86%)
Return to OR due to bleeding	5 (2.19%)
Stroke	3 (1.32%)
New onset hemodialysis	1 (0.44%)
New onset atrial fibrillation	17 (7.45%)
Phrenic nerve palsy	2 (0.88%)
Chylothorax	1 (0.44%)
Thoracic infection	4 (1.75%)
Early graft occlusion	4 (1.75%)
Early reoperation (sternotomy)	0
Drainage (mL)	434.5 ± 181.9
LOS (d) (mean ± SD)	5.5 ± 2.2
ICU stay (h) (mean ± SD)	1.5 ± 1.3

*n*: number; OR: operating room; LOS: length of stay; d: day; SD: standard deviation; ICU: intensive care unit; h: hour.

## Data Availability

This article’s data will be shared upon reasonable request to the corresponding author.
