# Correction: Demirsoy et al. The Feasibility and Early Results of Multivessel Minimally Invasive Coronary Artery Bypass Grafting for All Comers. *J. Clin. Med.* 2023, *12*, 5663

**DOI:** 10.3390/jcm15187144

**Published:** 2026-09-15

**Authors:** Ergun Demirsoy, Ilhan Mavioglu, Emre Dogan, Harun Gulmez, Ismet Dindar, Mustafa Kemal Erol

**Affiliations:** 1 Division of Cardiovascular Surgery, Sisli Kolan International Hospital, Kaptanpaşa Mahellesi Darulaceze Caddesi No 14, Sisli, 34384 Istanbul, Turkey; 2 Division of Cardiovascular Surgery, Private Practice, Sisli, 34394 Istanbul, Turkey; maviogluilhan@gmail.com; 3 Division of Cardiology, Sisli Kolan International Hospital, 34384 Istanbul, Turkey

**References**

To this publication [1] have been added one reference as reference 16. The citation in the main text is in the second paragraph of “2.1. Preoperative Preparation and Operative Technique”, as below:

The MACAB (off-pump, anaortic) technique has been described in detail in previously published articles [14–16].

With this correction, the order of some references has been adjusted accordingly. The authors state that the scientific conclusions are unaffected. This correction was approved by the Academic Editor. The original publication has also been updated.

Added Reference

16. Kyaruzi, M.; Gülmez, H.; Demirsoy, E. Can minimally invasive multivessel coronary revascularization be a routine approach? *Thorac. Cardiovasc. Surg.* **2023**, *71*, 455–461.
